# Editorial: Epigenetics, transcriptomics and epitranscriptomic – deal with old friends or new direction of regulation?

**DOI:** 10.3389/fpls.2025.1768516

**Published:** 2026-01-06

**Authors:** Jianping Wang, Szymon Kubala

**Affiliations:** 1 Agronomy Department, Plant Molecular and Cellular Biology Program, Plant Breeding Program, Genetics Institute, Institute of Food and Agriculture Sciences (IFAS), University of Florida, Gainesville, FL, United States; 2 Institute of Biochemistry and Biophysics Polish Academy of Science, Warsaw, Poland

**Keywords:** chromatin-RNA crosstalk, epigenetic regulation in plants, epitranscriptomic RNA modifications, plant gene regulatory networks, post-transcriptional gene regulation, RNA metabolism and RNA processing, transcriptome profiling

Gene regulation in plants is orchestrated through a multilayered network of molecular mechanisms encompassing chromatin organization, transcriptional control, RNA processing, RNA modification, and other processes. Epigenetics and transcriptomics have long provided foundational frameworks for understanding how gene expression is established and maintained. In recent years, however, increasing attention has been directed toward epitranscriptomic regulation, revealing an additional layer of control that substantially expand the regulatory landscape. This Research Topic, *Epigenetics, Transcriptomics, and Epitranscriptomic—Deal With Old Friends or New Direction of Regulation*, was conceived to explore how these regulatory dimensions operate independently and in concert, and to assess whether emerging RNA-centered mechanisms represent extensions of established paradigms or mark a genuine shift in our understanding of plant gene regulation.

The six articles brought together in this Topic span diverse biological contexts, including development, stress adaptation, disease resistance, and RNA metabolism, and encompass a range of experimental and analytical approaches. Collectively, they underscore a central theme: gene regulation in plants is inherently integrative, with epigenetic, transcriptional, and epitranscriptional processes forming interconnected regulatory networks rather than functioning as isolated layers.

Several contributions highlight the power of transcriptome-wide analyses in resolving complex regulatory processes. Genome-wide identification and expression profiling of the CesA/Csl gene family in Eucalyptus grandis provide a comprehensive view of transcriptional regulation underlying cell wall biosynthesis and secondary wall formation in a woody species. By integrating phylogenetic relationships, promoter cis-element analysis, and tissue-specific expression patterns, this study illustrates how transcriptional networks coordinate structural growth and stress-associated functions in long-lived plants (An et al.). A similar systems-level approach is applied to developmental regulation in tree peony, where transcriptome sequencing combined with endogenous hormone profiling is used to dissect the molecular basis of *in vitro* shoot apical dormancy during adventitious root formation. The findings demonstrate that dormancy is governed by coordinated modulation of hormone signaling pathways and metabolic gene expression, rather than by abscisic acid accumulation alone. The regulatory model proposed in this study provides mechanistic insight into dormancy control and offers practical implications for improving micropropagation efficiency in woody ornamentals (Yang et al.). Transcriptomic analysis also underpins the fine mapping and characterization of the Fusarium wilt resistance gene *Fob1(t)* in wax gourd. By combining bulked segregant analysis sequencing, gene expression profiling, and functional annotation, this work identifies an endochitinase gene as a key determinant of disease resistance. Beyond gene identification, the study illustrates how transcriptional regulation intersects with hormone signaling, cell wall modification, and defense-related metabolic pathways to shape resistance phenotypes, exemplifying the translational value of regulatory genomics in crop improvement (Li et al.). Complementing transcription-focused studies, this Research Topic places strong emphasis on post-transcriptional and epitranscriptomic regulation. A comprehensive review of RNA tailing by nucleotidyl transferase proteins synthesizes current knowledge on how non-templated nucleotide additions at RNA 3′ ends influence RNA stability, degradation, and function in plants. By detailing uridylation, adenylation, and other tailing events across diverse RNA substrates, the review positions RNA tailing as a central epitranscriptomic mechanism shaping gene expression during development, stress responses, and disease resistance (Chen et al.). The functional relevance of RNA-level regulation is further illustrated by original research revealing how ribosome binding to TAS (trans-acting siRNA) transcripts modulates ta-siRNA biogenesis in *Arabidopsis thaliana*. This study demonstrates that ribosomes play regulatory roles beyond translation, influencing transcript stability and processing in distinct ta-siRNA biogenesis pathways. These findings blur traditional boundaries between translation and RNA silencing and uncover unexpected crosstalk between translational machinery and post-transcriptional regulatory circuits (Wang et al.). Epigenetic regulation is represented in this Topic through a focused mini-review addressing plant responses to cadmium stress. This article synthesizes evidence that DNA methylation, histone modifications, and chromatin remodeling are integral to plant adaptation to heavy metal toxicity. By linking chromatin-level regulation with transcriptional and physiological responses, the review highlights the role of epigenetic plasticity in stress tolerance, stress memory, and potential applications in phytoremediation and crop breeding (Gao et al.).

Taken together, the contributions to this Research Topic reinforce several overarching concepts. First, plant gene regulation is intrinsically multi-layered and interconnected, with regulatory outcomes emerging from coordinated interactions among chromatin state, transcriptional activity, and RNA metabolism. Second, regulatory mechanisms are highly context dependent, varying across developmental stages, environmental conditions, and species. Third, epitranscriptomic and post-transcriptional processes—such as RNA tailing and small RNA biogenesis—are increasingly recognized as core components of regulatory networks rather than peripheral modifiers of transcription. In addressing the central question posed by this Topic, the assembled studies suggest that epigenetics and transcriptomics may indeed be viewed as well-established “old friends,” yet their integration with RNA-centered regulatory mechanisms represents a meaningful expansion of the gene regulatory framework. Rather than replacing existing paradigms, epitranscriptomic regulation enriches them by revealing how regulatory information is further processed, refined, and executed at the RNA level.

We anticipate that this Research Topic will stimulate further integrative research combining chromatin biology, transcriptomics, and RNA biology. Such approaches will be essential for developing comprehensive models of plant gene regulation and for translating fundamental insights into applications in agriculture, biotechnology, and environmental sustainability.

